# First-Principles-Based Optimized Design of Fluoride Electrolytes for Sodium-Ion Batteries

**DOI:** 10.3390/molecules27206949

**Published:** 2022-10-17

**Authors:** Shuhan Lu, Bingqian Wang, Panyu Zhang, Xiaoli Jiang, Xinxin Zhao, Lili Wang, Zhixiang Yin, Jianbao Wu

**Affiliations:** School of Mathematics, Physics and Statistics, Shanghai University of Engineering Science, 333 Longteng Road, Shanghai 201620, China

**Keywords:** ethylene carbonate, 1,2-dimethoxylethane, fluorination, first principles, sodium batteries

## Abstract

Because of the abundance and low cost of sodium, sodium-ion batteries (SIBs) are next-generation energy storage mediums. Furthermore, SIBs have become an alternative option for large-scale energy storage systems. Because the electrolyte is a critical component of SIBs, fluorination is performed to improve the cycling performance of electrolytes. Based on the first-principles study, we investigated the effects of the type, quantity, and relative position relationships of three fluorinated units, namely -CF_1_, -CF_2_, and -CF_3_, on the cyclic ester molecule ethylene carbonate (EC) and the linear ether molecule 1,2-dimethoxylethane (DME). The optimal fluorination was proposed for EC and DME by studying the bond length, highest occupied molecular orbital, lowest unoccupied lowest orbital, and other relevant parameters. The results revealed that for EC, the optimal fluorination is 4 F fluorination based on four -CF_1_ units; for DME, CF_3_CF_1_CF_1_-, CF_3_CF_2_CF_2_-, CF_3_CF_1_CF_2_CF_3_, and CF_3_CF_2_CF_2_CF_3_, four combinations of three -CF_1_, -CF_2_, and -CF_3_ units are optimal. The designed fluorinated EC and DME exhibited a wide electrochemical stability window and high ionic solvation ability, which overcomes the drawback of conventional solvents and can improve SIB cycling performance.

## 1. Introduction

Sodium-ion batteries (SIBs) exhibit considerable potential over lithium-ion batteries (LIBs) because of the high abundance and cost effectiveness of sodium [1,2]. A suitable electrolyte is crucial for high-performance SIBs. The electrolyte strongly influences the cycling performance of SIBs by affecting ion transport and regulating the electrochemical reaction behavior at the solid–liquid interface. The electrolyte is closely related to the formation of the solid–electrolyte interphase (SEI). Ponrouch et al. reported that an appropriate electrolyte can form an excellent SEI film on the anode and improve the cycling performance of the cell [3]. An optimized SEI can increase the kinetic stability of the electrolyte, prevent continuous reduction of the active substance during subsequent cycling, and allow ions to pass through the SEI. Based on the molecular orbital theory, Goodenough et al. [4] proposed that the kinetic stability of the electrolyte is closely related to the formation of SEI. The anode and cathode have the natural properties of being oxidant and reductant, respectively, which will lead to the redox of the electrolyte. If the stability of the electrolyte is to be maintained, it is necessary to introduce a proper SEI passivation layer, which makes the electrochemical potentials, μ_A_ and μ_C_, of the electrode fall within the window of the electrolyte. Electrolyte engineering solutions, such as electrolyte fluorination, electrolyte additives [5], optimized solvent/solute ratios [6,7], and high-concentration electrolytes [8,9], have been developed to improve the cycling performance of SIBs. The high-concentration electrolyte reduces the free solvent molecules in the solvated structure of metal ions, which results in the formation of SEI with mainly inorganic components to improve SIB-cycling performance. Qi et al. [10] reported a 1-octyl-3-methylimidazolium bis (trifluoromethylsulfonyl) imine ([OMIm] TFSI) additive, which can make a Li||Li symmetrical battery still have a capacity of 141.7 mAh/g after 200 cycles at a discharge rate of 0.5 C. Fluorinated ethylene carbonate (FEC) is typically used as a film-forming additive to promote the formation of LiF, which is an effective SEI passivation component [11,12]. He et al. [13] suggest that FEC regulates the formation of SEI and promotes the long-term performance of the corresponding battery. Besides ester-based electrolyte, Liang et al. [14] reported a fully coordinated ether-based electrolyte that when applied in graphite//NVPF full cells exhibited excellent rate performance and cycling stability. In the high-concentration electrolyte, realization of the solvation effect with metal ions is difficult; therefore, some additives (sulfolane [15], dimethyl carbonate [16]) are necessary for salt dissolution. However, these components typically produce continuous side reactions with solvent molecules or electrodes, which may decrease Coulombic efficiency (CE) [15,17].

The use of fluoride electrolytes [18,19,20], that is, different fluorinated units, has attracted considerable research attention to improve electrolyte performance by fluorinating solvent molecules in different ways. For example, Zhang et al. [21] proposed a high-performance SEI modifier for LIBs by analyzing the reaction mechanism, such as decomposition and bonding using the -CF_1_ group to gradually replace all the H atoms on the EC molecule. FEC has proved to promote NaF formation in SIBs [22]. Cui [20] et al. revealed that the linear molecule fluorinated 1,4-dimethoxybutane (FDMB) by the -CF_2_ group exhibits excellent cycling performance for lithium metal anodes. Furthermore, the team investigated the effect of the -CF_3_ group on the fluorination of the linear molecule ethyl methyl carbonate and provided unique insights [23]. Further research has demonstrated the improved Coulombic, voltage and energy efficiency of fluorination. As discussed above, based on 1,2-diethoxyethane, Cui et al. [20] prepared F4DEE and F5DEE by introducing the -CHF_2_ group and F3DEE and F6DEE by introducing the -CF_3_ group. The CE of DEE and fluorinated DEE were compared. The CE of DEE reached 99%; however, the CE of Li||Cu half-cell using 1.2 M LiFSI/F5DEE can reach 99.9%. Additionally, the CE could rapidly reach about 99.5% as the areal capacity was increased, which is one of the fastest. They further investigated the leakage current evolution of FDMB under high voltage, and the result shows a high voltage stability because of the similarity of leakage current and that of the conventional electrolyte. The anodic stability shows a trend of F5DEE ≅ F6DEE > F4DEE ≫ F3DEE, which is consistent with the trend of fluorination. Han et al. [24] reported an amphoteric additive, lithium fluoromalonato (difluoro) borate (LiFMDFB), which not only improved the interfacial stability of Li-rich positive electrodes/high-capacity Si-embedded negative electrodes but also had an excellent cycle performance in the SGC‖Li_1_._17_Ni_0_._17_Mn_0_._5_Co_0_._17_O_2_ full cell, which maintained a high CE (99.5% after 100 c cycles) and reached an energy density of 400 Wh/kg after FEC was added. The fluorinated electrolyte was also used in the K-ion battery but not only in the SIB and the LIB. Hao et al. [25] introduced a retentive solvation configuration, weakened anion-coordination, and non-solvation fluorinated ether electrolyte; with this electrolyte, they reported optimal inorganic/organic components, providing a promising application prospect.

As discussed above, the effect of fluorination, including the aforementioned units on cyclic carbonate molecules [26,27], linear ethers [28], and other molecules on the LIB electrolytes, were investigated at the same time; the first-principles calculation was performed to study fluoride electrolytes for sodium-ion batteries [29,30,31,32]. However, limited studies have focused on the systematic design of fluoride electrolytes for SIBs based on the -CF_1_, -CF_2_, and -CF_3_ units for the two types of solvent molecules.

In this study, the first-principles study was performed to investigate two types of solvent molecules: the first molecule is the cyclic ester molecule based on EC, and the second molecule is the linear ether molecule based on 1,2-dimethoxylethane (DME). EC and DME are typical representatives of cyclic ester and linear ether solvents in current LIB and SIB electrolyte systems, respectively. By adjusting the types, numbers, and positions of the fluorinated units, -CF_1_, -CF_2_, -CF_3_, we found that: (1) the fluorinated EC, -CF_1_, and -CF_2_ groups exhibit a strong modulating effect on the cyclic performance of EC, and -CF_3_ is not used because of the saturation of EC fluorination. By analyzing the highest occupied molecular orbital (HOMO) to lowest unoccupied molecular orbital (LUMO), EC fluorinated by two -CF_2_ exhibited an energy gap up to 9.1 eV and high electrochemical stability. Furthermore, the evaluation of the bond length and restrained electrostatic potential (RESP) charge verified the improvement of FEC by deep fluorination. (2) For fluorinated DME, -CF_1_, -CF_2_, and -CF_3_ can affect its cycling performance, but -CF_3_ exhibits the strongest effect. In addition to the type, the number and position of those units can also considerably affect fluorinated DME. The energy gap of fully fluorinated DME reaches 10.58 eV, which implies a higher electrochemical stability window. The study of electron affinity potential implies the improved effect of strong fluorination on DME. However, the properties of fluorinated DME do not change unidirectionally with the increase in F atoms. Therefore, certain weaker strengths of fluorination can achieve properties similar to deep fluorination through specific combinations of units. Two design methods of fluorinations exhibit the strongest modification effects on DME: (1) fluorination with two identical units at two sites near -CF_3_ when three fluorination sites exist and (2) deep fluorination with four sites. The calculation conclusion reveals how the fluorination units affect the EC and DME through type, number, and position. Finally, we designed five optimized combinations of units with superior cycling performance. In general, based on the skeletons of EC/DME and by fluorinating with -CF_1_, -CF_2_, -CF_3_, we investigated the specific properties of fluorinated solvents, and five preferred fluorination methods were provided, which presents a novel paradigm for electrolyte modification.

## 2. Results and Discussion

### 2.1. Fluorination of EC

**Figure 1:**
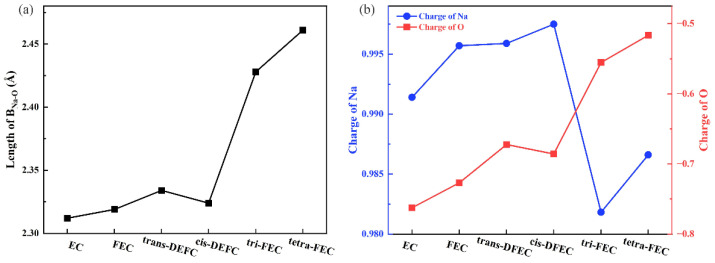
(**a**) Length of B_Na-O_, where the O atom belongs to the carbonyl group. (**b**) Atom charge of O and Na, where the O atom belongs to carbonyl group.

EC, as a solvent, exhibits excellent solubility for salts and is a widely used electrolyte solvent [33]. The structure of the system composed of EC and Na-ion was optimized by considering the solvation effect and van der Waals force. The Na bond can reduce LUMO by changing the ratio of C atomic orbital in LUMO or the composition of LUMO, which not only promotes the decomposition of electrolyte, but also makes the charge transfer between solvent molecules and Na ions. Under the action of various factors, the stability of [Na^+^-solutions] complex will also change; that is, it changes the solvation ability of the solvent. Thus, The Na bond is often considered as the judgement standard for solvation ability for its important interaction in SIB, such as the Li bond and the hydrogen bond in LIB [34,35], and it has close relationships with B_Na-O_, etc. The distance of Na and O in this paper is smaller than that of the average value in the [Na^+^-EC] solvation sheath (2.359 Å) [36], which exhibits an absorption effect in Na^+^ and O atom, forming a B_Na-O_ bond with a bond length of 2.312 Å. As displayed in Figure 1a, the carbonyl oxygen in the fluorinated EC molecule exhibited a considerable adsorption effect on Na-ion, but the B_Na-O_ bond length increased with the strengthening of fluorination and reached 2.461 Å when all the H of EC were fluorinated. This length is already considerably larger than the bond length of carbonyl oxygen to Na-ion in EC. The difference between the B_Na-O_ bond lengths of trans-DFEC and cis-DFEC is only 0.011 Å, but both lengths are larger than the B_Na-O_ bond lengths of the EC and FEC systems. This result indicates that both configurations of DFEC exhibit similar strengths for Na-ion adsorption. However, both configurations are weaker than EC and FEC because of stronger fluorination. Furthermore, the increase in the bond length of the tri-FEC system was significant (~0.1 Å compared with that of DFEC), which is considerably larger than the increase from 0 F to 1 F and 1 F to 2 F substitution. Thus, the change in the bond length with the number of F atoms is not strictly linear. The change in the B_Na-O_ bond length reveals that the attraction ability of FEC molecules to Na-ion gradually becomes weaker, and this trend is especially significant under 3 F and 4 F fluorination. Thus, with the weakening of the force between solvent molecules and metal ions, the solvation ability of the solvent become weaker, and the Na-ions are more likely to be extracted from the solvated sheath and embedded into the electrode, which is conducive to the progress of the reaction [37].

Similar conclusions can be drawn in the charge transfer of these systems. We conjectured that the charge leads to the change in the electrostatic force and thus the bond length. We calculated the RESP charge of the carbonyl O in the six molecules of EC and fluorinated EC and the Na-ion bonded to it, as displayed in Figure 1b. The change in the charge of the carbonyl O atom exhibits a similar trend to the change in the bond length; that is, the charge on the carbonyl O becomes smaller (more positive) as the fluorination proceeds, and the carbonyl O of trans-DFEC and cis-DFEC carries almost the same amount of charge (−0.672, −0.686). Similarly, the carbonyl oxygen in tri-FEC exhibits the largest amount of variation. The weakened charge implies a weakened electrostatic force, which confirms the variation of the B_Na-O_ length. By contrast, a more positive charge variates the binding effect of anion in Na-salt and regulates the solvation structure, which results in the formation of NaF-rich SEI. Compared with the significant charge change on the carbonyl oxygen, the charge change of the Na-ion is considerably smaller, with the maximum difference of only 0.016 (the charge difference between Na-ion and tri-FEC/cis-DFEC), which indicates that the fluorination of EC molecules is less likely to change the charge distribution of adsorbed Na-ion.

**Figure 2:**
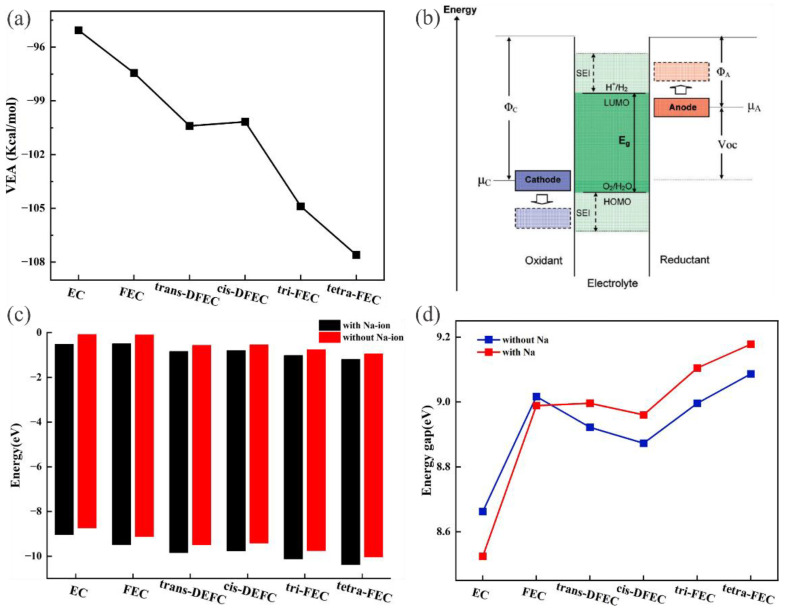
Vertical electron affinity (VEA) of EC and fluorinated EC (**a**). Schematic of ESW (**b**), Φ_A_ and Φ_C_ are the anode and cathode work functions, E_g_ is the ESW [4]. HOMO–LUMO of EC and fluorinated EC when Na-ion were added (**c**). HOMO–LUMO energy gap of EC and fluorinated EC when Na-ion were added (**d**).

The change in the atomic charge is closely related to the migration of electrons. Considering that the electron affinity potential can describe the thermodynamic change before and after accepting electrons, to understand the mechanism of charge change, we investigated the effect of fluorination on the vertical electron affinity (VEA) of the system. VEA is defined as the energy released by a molecule after gaining an electron (being reduced), and it characterizes the tendency of the molecule to absorb electrons. VEA can be calculated by the following equation:

(1) VEA=E(N+1)−E(N),

where E(N + 1) denotes the single-point energy of the system after obtaining one electron, and E(N) denotes the single-point energy of the system before receiving an electron. We first optimized the structures before gaining electrons and calculated their single-point energies, which were used to calculate the single-point energy after gaining one electron. The VEA was obtained by the numerical difference between these two energies (Figure 2a). Comparing the effect of fluorination on VEA, the trend of VEA remained highly consistent with the changing trends of the B_Na-O_ length and charge, which reflects a decrease (more negative) with the deepening of fluorination, whereas trans-DFEC and cis-DFEC exhibited similar VEA values.

HOMO exhibits electron donor characteristics, and LUMO exhibits electron acceptor characteristics with strong affinity for electrons. Therefore, higher HOMO energy levels indicate easier loss of electrons and poorer oxidative stability, and higher LUMO energy levels indicate a difficult gain of electrons and superior reductive stability. We expect the HOMO–LUMO trend to be similar to VEA. The HOMO–LUMO of EC compared with fluorinated EC was calculated. Various degrees of reduction were observed in HOMO–LUMO energy levels because of the fluorination in the Figure 2c. For solvent molecules, the reduced HOMO–LUMO energy level contributed to the decomposition of the anion, which generated anion-derived inorganic SEI components [38]. To simulate the solvation environment in real electrolytes, we introduced the Na-ion to form coordination with solvent molecules and subsequently calculated the HOMO–LUMO energy levels of metal salt ion solutions (Figure 2c). With the dissolution of Na-ion, the HOMO–LUMO energy levels of all six solvent–solute systems were lower than the HOMO–LUMO energy level of pure solvent, which could be attributed to the change in electronic states when the solvent molecules form [Na-solvent]^+^ complexes with the solute [39]. Therefore, the HOMO–LUMO of fluorinated EC with Na-ion solutions were considerably lowered by the combined effect of fluorination and metal salt ions, which induced the formation of protective SEI.

A wide electrochemical stability window (ESW) is desired to provide a stable operating environment for the cell. Goodenough proposed that the ESW of an electrolyte can be inferred from its relative HOMO–LUMO difference (Figure 2b) [4,40,41] as follows:

(2) ESW=E(LUMO)−E(HOMO),

where E(LUMO) denotes the energy of LUMO and E(HOMO) denotes the energy of the HOMO. The increased HOMO–LUMO energy gap can effectively prevent the electron transfer from the electrode to the electrolyte component through the tunneling effect and avoid the continuous decomposition of the electrolyte at a low electric potential [42]. We compared the HOMO–LUMO energy gap of EC under various levels of fluorination (Figure 2d). The results revealed that fluorination increased the energy gap of EC and reached a maximum (9.1 eV) in the case of 4 F fluorination. However, unlike the changes in the bond length, charge, and VAE, the HOMO–LUMO energy gap of FEC is not only considerably larger than that of EC but is even larger than that of DFEC and tri-FEC and only slightly smaller than that of tetra-FEC. To reflect the real-world solution environment, we compared the energy gaps of the six systems in the presence of Na-ion (Figure 2d), which revealed that the presence of Na-ion had distinct effects on the six systems. The Na-ion decreased the energy gap of EC and considerably increased the energy gaps of DFEC, tri-FEC, and tetra-FEC but had almost no effect on the energy gap of FEC. In the actual solution environment, the weakly fluorinated FEC did not exhibit a high ESW, whereas the ESW of DFEC and tri-FEC remained lower than that of the strongly fluorinated tetra-FEC. By contrast, tetra-FEC exhibited the widest ESW.

Numerous studies have investigated EC and FEC synthesis and their applications in SIBs [43,44]. However, limited studies have reported the synthesis of tetra-FEC and its composition in electrolyte systems. Considering previous studies have investigated balanced and excellent performance of tetra-FEC in SIB systems, tetra-FEC is a highly promising solvent molecule that provides a novel approach for SIB electrolyte optimization strategies. A similar conclusion was verified in LIBs. Zhang [21] et al. confirmed the superiority of tetra-FEC applied to LIBs to produce a homogeneous, denser LiF-containing SEI, which renders tetra-FEC a high-performing SEI modifier. Thus, tetra-FEC is an optimal choice for electrolytes in both SIBs and LIBs. The calculated electrostatic potential mapping of the [Na^+^-Tetra-FEC] complex is shown in Supplementary Materials (Figure S1); its atom coordinates are also supplemented.

### 2.2. 1,2-Dimethoxylethane Fluorination

DME, which stands for 1,2-dimethoxylethane, is a linear ether solvent molecule typically used in SIB electrolytes. The ether-based solvent has a specialized solvated structure, and the electrolyte formed with Na-ion exhibits several characteristics.

1. Compared with carbonate ester solvents, sodium–ether complexes exhibit high LUMO, low solvation energy, and good stability in electrochemical reactions.
2. Compared with carbonate ester solvents, ether-based solvents exhibit high reductive stability, which can inhibit dissolution during discharge and form a thin SEI layer. SEI formation typically occurs during the first few charge/discharge cycles of the battery, with sodium salts decomposing preferentially into the inorganic components of the inner layer and ether solvents decomposing into the organic components of the outer layer. An appropriately designed electrolyte facilitates the formation of NaF during the decomposition process and effectively protects the SEI.
3. Ether solvents are more compatible with electrodes because of saturated bonds, which reduces the occurrence of side reactions and enhances cycling performance.

Despite these advantages, ethers exhibit poor oxidative stability and using them in high-voltage battery systems is difficult. In this study, we applied -CF_1_, -CF_2_, and -CF_3_ to design fluorinated DME.

**Figure 3:**
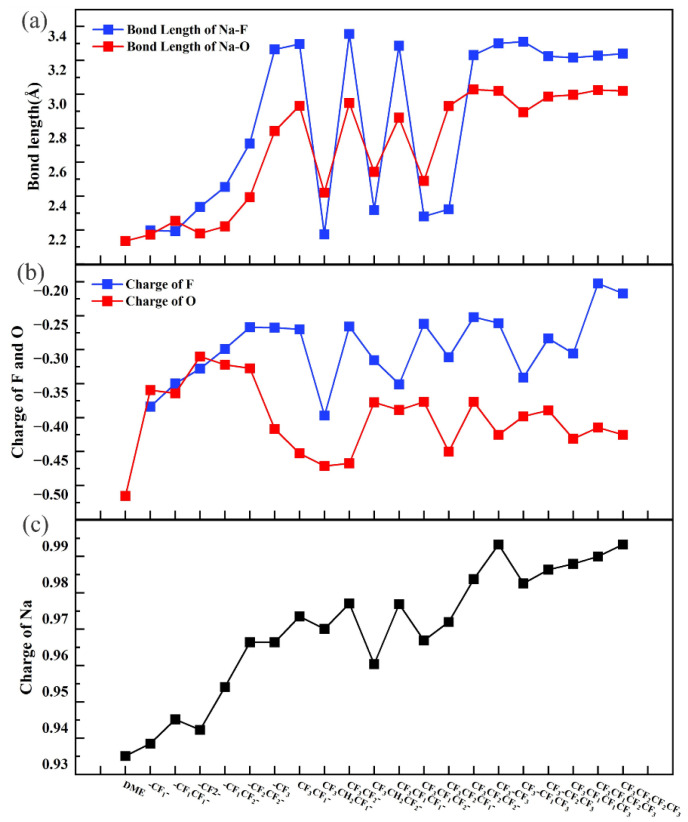
Bond length of B_Na-O_ and B_Na-F_ (**a**). Atom charge of O, F (**b**) and Na-ion (**c**).

For the consistency of results, as in EC, we considered the van der Waals force under the solvation effect, optimized the structure of the electrolyte system of DME, fluorinated DME with Na-ion, calculated the B_Na-O_ bond length, and measured the distance between Na and F. Because multiple Na-ions or F atoms exist, we considered the smallest (Figure 3a). The results revealed that the Na-ions bond with O (bond lengths between 2.336 and 3.229 Å). Considering that the atomic radius of F is smaller than that of the Na atom, but the distance between Na and F (between 2.375 and 3.556 Å) is only slightly larger than the Na-O bond length, the Na-ion also bonds with the F atom in the fluorinated DME with some strength as well. In -CF_1_/-CF_2_ units only, the maximum number of F atoms can reach 4 (1 F–4 F), and the bond lengths of B_Na-O_ and B_Na-F_ are maximum at 2.594 and 2.911 Å, respectively. However, the bond length of the -CF_1_CF_2_- combination, which is composed of -CF_1_ and -CF_2_ units only is markedly smaller than that of -CF_3_, which is even larger than that of 4 F fluorinated -CF_2_CF_2_-. This phenomenon indicates that the bond length increase induced by the -CF_3_ group is considerably larger than those of the -CF_1_ and -CF_2_ units. The bond length change in the interval of 1 F–4 F can be described as a “unidirectional increase.” After one site is fluorinated with -CF_3_, if -CF_1_/CF_2_ groups are introduced (4 F–6 F) simultaneously, the bond lengths exhibit “oscillating” trends. If only one -CF_1_ or -CF_2_ are introduced, that is, two fluorination sites, the bond lengths of the CF_3_CF_1_- and CF_3_CF_2_- combinations are longer than those of the CF_3_CH_2_CF_1_- and CF_3_CH_2_CF_2_- combinations. In the case of only two fluorinated units (one of which is -CF_3_), the introduction of a fluorinated group at the site adjacent to -CF_3_ increases the B_Na-O_ bond length considerably. This result is also applicable to the B_Na-F_ bond length. If two -CF_1_/-CF_2_ units are introduced with one-CF_3_, that is, with three fluorinated sites, the bond lengths of CF_3_CF_1_CF_1_- and CF_3_CF_2_CF_2_- are similar, and the difference compared with CF_3_CF_1_- and CF_3_CF_2_- (two fluorinated sites) is not large but greater than CF_3_CF_1_CF_2_- and CF_3_CF_2_CF_1_-. Then, in the case of three fluorinated sites (with only one -CF_3_ group), the use of different units with -CF_1_/-CF_2_ to fluorinate the C2/C3 site considerably reduces the B_Na-O_ bond length. Furthermore, the B_Na-F_ bond length changes almost synchronously with it in both intervals. Two -CF_3_ units should be introduced if the fluorination intensity is increased. Regardless of -CF_1_/-CF_2_ units between the two -CF_3_ units, the B_Na-O_ bond length no longer increases considerably with fluorination. In this interval (6 F–10 F), the change in the bond length can be described as “convergent”. The bond lengths of B_Na-F_ also exhibit a high degree of agreement with the bond lengths of B_Na-O_.

The variation of bond lengths is highly correlated with types, numbers, and relative positions of those units and also with the number of F atoms, showing “unidirectional variational”, “oscillating”, and “convergent” tendencies, which could also be reflected to some extent in subsequent studies.

As in the case of fluorinated EC, we explained why the variations of the B_Na-O_ and B_Na-F_ bond lengths exhibited such a pattern by the atomic charges of Na, O, and F. Notably, the charge over O does not tend to decrease markedly (more positive) but gradually converges to a value between the maximum (−0.465 e) and the minimum (−0.260 e) with the increasing number of F atoms (Figure 3b). The un-fluorinated DME exhibited the largest O atom charge (−0.465 e) and the strongest electrostatic force with Na-ion. Therefore, the shortest Na-O bond length of 2.336 Å was achieved. With the exception of this result, no clear correspondence was observed between the change in the charge and bond length of B_Na-O_. For example, in the “oscillating” interval, the atomic charges of O in CF_3_CF_1_-, CF_3_CF_2_-, CF_3_CF_1_CF_1_-, and CF_3_CF_2_CF_2_- were not considerably larger than those in CF_3_CH_2_CF_1_-, CF_3_CH_2_CF_2_-, CF_3_CF_1_CF_2_-, and CF_3_CF_2_CF_1_-. Although the charge change of O could also be described as “unidirectional variational”, “oscillating”, and “convergent”, this description is not as precise as that of the variation in bond lengths. The charge change on F does not strictly correspond to the change in the B_Na-F_ bond length, but a large decrease occurs with fluorination and exhibits vague tendencies of “unidirectional variational”, “oscillating”, and “convergent”.

The atomic charge of the Na-ion was calculated (Figure 3c), and although it exhibits a similar trend to the bond length, it is not sufficient to considerably affect the bond length, considering that the change is only 0.058 e. Thus, the fluorination dose decreased the atom charge of O and F and weakened the electrostatic interaction of Na-O and Na-F compared with DME. Therefore, the lengths of B_Na-O_ and B_Na-F_ increased considerably. Furthermore, fluorination promoted charge transfer from O to F, which could explain the reduced charge of O. The transfer regulated the combination of solvent and solute, altered the solvation of Na-ion, and contributed to the formation of NaF-rich SEI. A parallel conclusion is drawn in the subsequent discussion.

**Figure 4:**
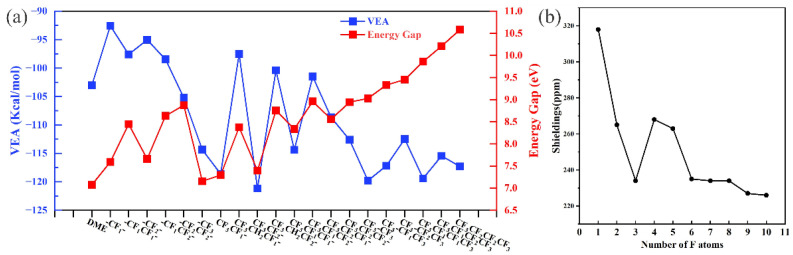
VEA and energy gap of HOMO–LUMO (**a**) and the relationship between absorption peaks (NMR) with F numbers (**b**).

Considering that the variation of VEA during the fluorination of EC is highly consistent with the variation of the atomic charge, this consistency could carry over to fluorinated DME. The same method was used to calculate the variation of VEA with fluorination (Figure 4a). Notably, the VEA did not maintain the same trend of atomic charge but was highly consistent with the change in the bond length. The VEA of -CF_3_ is considerably smaller (more negative) than that of -CF_1_/-CF_2_, and only the VEA of -CF_3_ is lower than that of DME. This result indicates that the -CF_3_ group can considerably increase the reductive activity of the molecule/compound. For the “oscillating” interval, in the case of two fluorination sites, CF_3_CF_1_- and CF_3_CF_2_- are considerably smaller than CF_3_CH_2_CF_1_- and CF_3_CH_2_CF_2_- and even slightly lower than that in deep fluorination. In the case of three fluorination sites, CF_3_CF_1_CF_1_- and CF_3_CF_2_CF_2_- are lower than CF_3_CF_1_CF_2_- and CF_3_CF_2_CF_1_-. The VEA of three fluorinated sites may not be higher than the combinations of two fluorinated sites, which is not consistent with the change in the bond length. After already being fluorinated with two -CF_3_, if the C2/C3 is fluorinated again, it produces some oscillations, but the amplitude reduces considerably. In terms of trend, reducing the VEA by 10 F fluorination is difficult. Apparently, fluorination increases the reductive activity of the solvent molecules, whereas fewer F atoms can also reduce the VEA of the system in specific combinations.

We speculated that fluorinated DME should exhibit a wider ESW and calculated the HOMO–LUMO energy gap of fluorinated DME with the Na-ion system under the same environment (Figure 4a). The results revealed that the energy gap is already larger than the fluorinated EC when the fluorination degree reached 3 F or more. The change in the energy gap differs from the existing pattern, and “unidirectional variational”, “oscillating”, and “convergent” were not observed during the fluorination process. The oscillation of the energy gap disappeared gradually until the fluorination degree was above 6 F and started to increase gradually, reaching the maximum value at 10 F fluorination. In the case of strong fluorination above 6 F, the fully fluorinated DME exhibited the largest ESW, but in the case of weak fluorination below 6 F, -CF_2_CF_2_- and CF_3_CF_1_CF_2_- exhibited large HOMO–LUMO energy gaps.

The shift in the absorption peak in NMR spectral of ^19^F was recorded with the number of F atoms (Figure 4b) to assess the effect of fluorination on the solvent molecule and the anion–cation bonding environment of the salt. An upfield shift was observed in the NMR absorption peak of ^19^F with the increase in the number of F atoms. This result implied a strong ionic solvation and contributed to the enhanced pairing of Na-ion with the anion of sodium salt. Furthermore, the shift intensity fluctuated to some extent in the fluorinated interval from 4 F to 6 F, whereas the shift intensity of fluorination from 6 F to 10 F (Δ = 9 ppm) was considerably smaller than that from 1 F to 3 F (Δ = 82 ppm) and almost ceased to change. The shift in the absorption peak revealed the tendencies of “unidirectional variational”, “oscillating”, and “convergent.”

Finally, the difficulty of NaF formation in SEI was investigated because it considerably affects electrolyte performance. More NaF can restrain the growth of dendrite and reduce the loss of electrolyte active material during charge/discharge cycles [45]. In this study, the shorter the B_Na-F_ bond length is, the longer the B_C-F_ bond length is, and the larger the B_Na-O_ and B_Na-F_ bond angles α_O-Na-F_ are. Thus, this phenomenon implies a higher inclination of NaF formation (Table 1). Unexpectedly, the combination of lower degree fluorination, -CF_1_, and -CF_1_CF_1_- appeared conducive for NaF formation, and a higher degree of fluorination results in an opposite trend. Although the lower degree of fluorination has the tendency for NaF formation, the number of F atoms of a single-solvent molecule is considerably less than that of the strongly fluorinated molecule, which may generate more NaF components during the electrochemical cycle. This result proves that fluorination could regulate charge transfer and promote NaF formation. Furthermore, based on the previous discussion, weakly fluorinated solvents were considerably weaker than strongly fluorinated solvents in terms of solvation ability, reductive activity, ESW, and other factors. Therefore, deep fluorination, such as CF_3_CF_1_CF_1_-, CF_3_CF_2_CF_2_-, CF_3_CF_1_CF_2_CF_3_, and CF_3_CF_2_CF_2_CF_3_, remains the best fluorination strategy.

**Figure 5:**
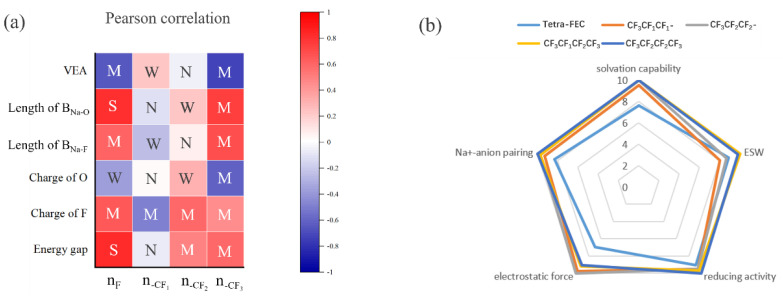
Pearson correlation analysis implemented in the heat map (**a**). Radar plot evaluating five solvents. The ratings between 1 and 10 are given according to the measured performance of each solvent. A rating of 10 represents strongest solvation capability, largest ESW, highest reductive activity, least electrostatic force, and strongest Na-anion pairing, whereas 0 denotes the opposite (**b**).

Because of the complexity of the problem, Pearson correlation (PC) analysis [46] was used to understand the close relationship among properties and the types, numbers, and positions of the three units. The Pearson correction coefficient is between −1 and 1, with −1 indicating a perfectly negative linear correlation, 1 indicating a perfectly positive linear correlation, and 0 indicating no linear relationship at all. The Pearson correction coefficient is calculated as follows:

(3) $$PC_{xy}=\frac{\sum_{i=1}^{n}\left(x_{i}-\bar{x}\right)\left(y_{i}-\bar{y}\right)}{\sqrt{\sum_{i=1}^{n}{\left(x_{i}-\bar{x}\right)}^{2}}\sqrt{\sum_{i=1}^{n}{\left(y_{i}-\bar{y}\right)}^{2}}},$$

where *x* and *y* denote two random variables, *i* denotes the index in the regression sample, and *n* denotes the number of samples. In this study, we define four correlation strengths “strong”, “medium”, “weak”, and “negligible”, corresponding to the cases when the absolute values of the PC coefficients fall in the intervals (0.8, 1.0), (0.4, 0.8), (0.2, 0.4), and (0.0, 0.2), respectively (abbreviated as S, M, W, and N, respectively) to improve readability. Figure 5a displays the PC coefficients of several variables in the heat map, with the labels “n_F_”, “$n_{{\text{-CF}}_{1}}$”, “$n_{{\text{-CF}}_{2}}$”, and “$n_{{\text{-CF}}_{3}}$” denoting the number of F atoms in each of the three units. “VEA”, “Length of B_Na-O_”, “length of B_Na-F_”, “charge of O”, “charge of F”, and “energy gap”, as signified by their literal meanings, denote variables closely related to F atoms and units. Finally, PC analysis revealed that the VEA, bond length, energy gap, and charge of F all exhibited strong correlation with the number of F atoms, among which the correlation strength of the B_Na-O_ bond length is “S”, which is markedly stronger than that of B_Na-F_ bond length “M”, indicating that fluorination has a stronger effect on enhancement of solvation ability than the inhibition of NaF formation. Among the three units, the modification effect of -CF_3_ of the aforementioned six variables is considerably greater than that of -CF_1_ and -CF_2_. As discussed in the previous section on the bond length, the amount of change in the bond length after the introduction of -CF_3_ is considerably greater than that of any combination of -CF_1_ and -CF_2_.

**Figure 6:**
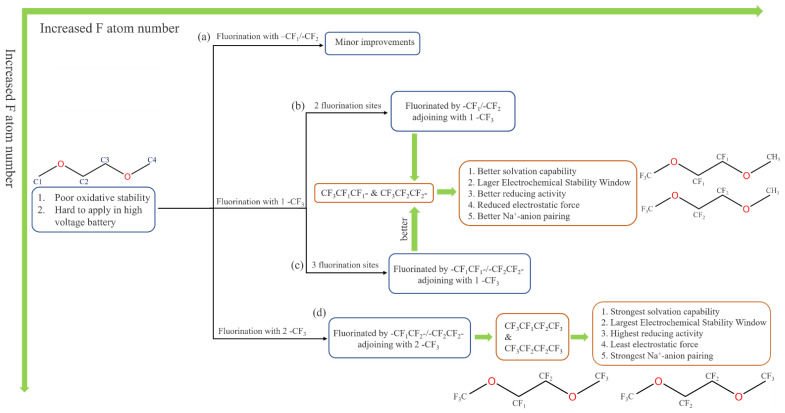
Fluorination logic flow. Fluorination with -CF_1_/-CF_2_ (**a**), fluorination with two fluorination sites (**b**), fluorination with three fluorination sites (**c**), fluorination with two -CF_3_ (**d**).

Combined with the previous discussion on fluorination sites, -CF_1_ and -CF_2_ exhibited limited effects on fluorinated DME. Without the participation of -CF_3_, improving the properties of DME with the other two units is difficult (Figure 6a). By contrast, although -CF_3_ alone exhibited a considerable effect, it can be regulated if -CF_1_/-CF_2_ is “properly” introduced with the participation of -CF_3_, where “properly” denotes: (1) If one -CF_3_ is introduced, 1 F/2 F fluorination occurs at the adjacent sites of -CF_3_ in the case of only two fluorination sites (Figure 6b), and fluorination occurs with the same moiety(-CF_1_/-CF_2_) at the C2 and C3 sites in the case of three fluorination sites (Figure 6c). When considering the HOMO–LUMO energy gap and upfield shift, CF_3_CF_1_CF_1_- and CF_3_CF_2_CF_2_- with three fluorination sites synthesize better. (2) If two -CF_3_ units were introduced, the 6F fluorination was reached at this point, and although its performance appeared to be leveling off, the maximum fluorinated CF_3_CF_1_CF_2_CF_3_ and CF_3_CF_2_CF_2_CF_3_ were the optimal solutions with balanced performance (Figure 6d). In conclusion, as expressed in the previous section, we kept the skeleton of EC and alkyl chains unchanged and explored the effect of the method when the three groups are suspended (type, number, and position) on the bond length, charge, and other structural properties. For EC, a fully fluorinated tetra-FEC with two -CF_2_ groups has the best performance; for DME, -CF_3_ can substantially improve its performance, followed by -CF_2_. The number of F atoms is equally important in synergy with the groups, so two -CF3 plus at least one -CF_2_ can greatly optimize the DME molecule, and one -CF_3_ plus two adjacent identical groups is slightly inferior. The calculated electrostatic potential mapping of the four optimal [Na^+^-fluorinated DME] complexes are shown in Supplementary Materials (Figure S1); their atom coordinates are also supplemented.

Finally, the five solvents discussed in this study were evaluated from five perspectives (Figure 5b), all which were optimal solutions with balanced performances among the fluorinated cyclic and linear solvents; the fully fluorinated tetra-FEC was the best overall choice among the fluorinated ECs in terms of solvation ability for Na-ion, pairing ability with anions, electrostatic adsorption, reductive activity, and ESW. CF_3_CF_1_CF_1_- and CF_3_CF_2_CF_2_- were superior to tetra-FEC in all aspects except for the ESW; the ESW of CF_3_CF_1_CF_2_CF_3_/CF_3_CF_2_CF_2_CF_3_ was greater than that of CF_3_CF_1_CF_1_-/CF_3_CF_2_CF_2_, but other properties did not differ considerably. Therefore, the appropriate solvent molecule could be selected according to the actual needs.

## 3. Materials and Methods

In this article, we kept EC molecules and the alkyl chain skeletons of DME unchanged. For EC, due to the saturation of the molecule, we suspended the -CF_1_ and -CF_2_ groups on the two C atoms, respectively, to produce five fluorinated EC molecules; for DME, we suspended -CF_3_ on the two terminal C atoms and -CF_1_/-CF_2_ on the middle 2 C atoms to produce 20 fluorinated DME molecules and calculated the related properties after optimizing their structures.

### 3.1. Design Logic Flow of Fluorinated EC

**Figure 7:**
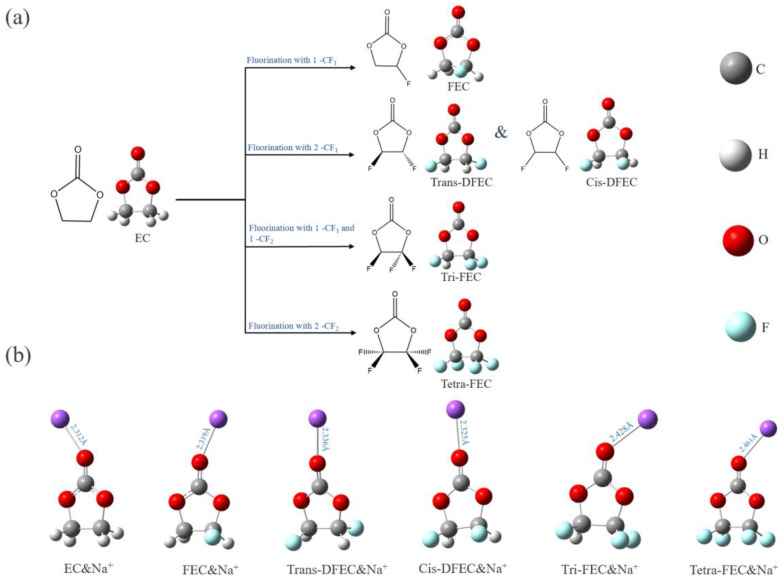
Design logic flow of EC fluorination (**a**) and the optimized structure of Na-ion with those molecules (**b**).

EC is a cyclic ester molecule typically used in electrolyte solvents. In this study, first, five configurations of fluorinated EC, namely EC, FEC, trans-difluoroethylene carbonates (trans-DFEC), cis-difluoroethylene carbonates (cis-DFEC), trifluoroethylene carbonate (tri-FEC), and tetrafluoroethylene carbonate (tetra-FEC) were developed as solvent molecules. Because of the symmetry of EC molecules, two configurations were obtained when fluorinated with two -CF_1_ to obtain DFEC: trans (two F atoms on each side) and cis (two F atoms on the same side), respectively, as illustrated in Figure 7a. Next, the RESP charge, HOMO–LUMO, and electron affinity potentials of EC and fluorinated EC were calculated. Na-ion was introduced, and the electrolyte system consisting of EC and fluorinated EC molecules with Na-ion (Figure 7b) was constructed. The properties of the six systems were investigated and the differences before and after the introduction of Na-ion were compared. All structures were optimized, and no imaginary frequencies were observed.

### 3.2. Design Logic Flow of Fluorinated DME

**Figure 8:**
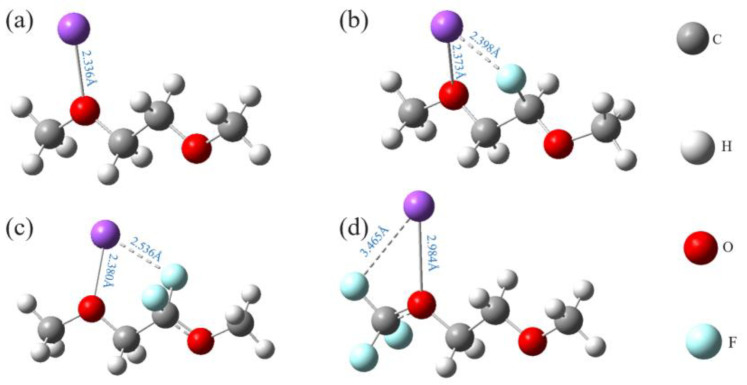
Structures of Na-ion and DME (**a**), fluorinated DME by -CF_1_ (**b**), -CF_2_ (**c**), -CF_3_ (**d**).

To improve the cycling performance of SIBs, three fluorinated units, -CF_1_, -CF_2_, and -CF_3_, of various combinations were suspended on the alkyl chain of DME. To ensure the solvated ability of Na-ion in ether solvents, we avoided directly connecting the fluorinated units to O atoms but suspended them to C atoms on the alkyl chain. Na-ion can combine with O atoms to form solvated sheaths, and fluorination decreases the charge on O and enhances the solvated ability of DME [47]. For DME fluorinated with one of the -CF_1_, -CF_2_, and -CF_3_ units alone, the structures of those fluorinated DME with Na-ion systems are displayed in Figure 8. The B_Na-O_, B_Na-F_, and B_C-F_ bond lengths and B_Na-O_ and B_Na-F_ bond angles of the remaining fluorinated DME configurations are listed in Table 1. By varying the combinations of the three units, we systematically investigated the effects of the group types, numbers, positions, and the number of F atoms on the subsequent properties of DME, such as oxidative stability and solvation ability.

**Figure 9:**
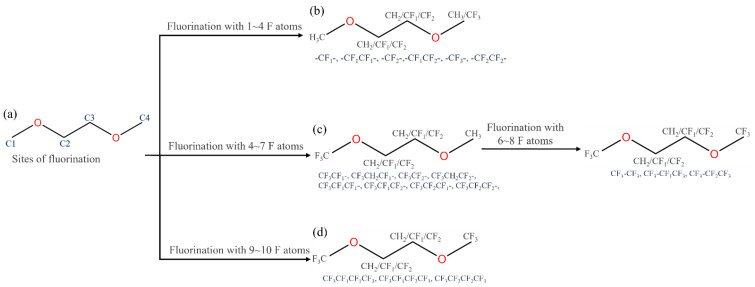
Fluorinated sites of DME (**a**) and detailed fluorination of DME with 0–4 F (**b**), 4–8 F (**c**), and 9–10 F (**d**).

The specific fluorination methods are: (1) in the alkyl chain, four fluorination sites C1, C2, C3, and C4 exist (Figure 9a). First, at the C1 site, fluorination was not performed, and at the C2/C3 and C4 sites, fluorination was performed by using -CF_1_/-CF_2_ and -CF_3_. Thus, 1 F–4 F fluorinated DME molecules were obtained (Figure 9b). (2) At the C1 site, fluorination was performed by using -CF_3_, then fluorinating with -CF_1_/-CF_2_ at the C2 and C3 sites, respectively, while suspending the C4 site with -CH_3_ moiety, which facilitated investigation of the effect of the fluorination site spacing on the solvent molecule. Based on this result, the C4 site was fluorinated with -CF_3_ afterward, at which point, three sites were fluorinated, which facilitated the subsequent study of the effect fluorinated sites number on solvent molecules. Thus, 4 F–8 F fluorinated DME molecules were obtained (Figure 9c). (3) Finally, a deeper fluorination was performed with -CF_1_/-CF_2_ to obtain 9 F–10 F fluorinated DME molecules at which point the number of fluorination sites reached four (Figure 9d).

### 3.3. Computational Setups

In this study, geometry optimization was performed using the B3LYP/6-311+g(d,p) basis set based on DFT calculations. The calculation levels of electron affinity energy, single-point energy, and Gibbs free energy were consistent with the geometry optimization, and all energy calculations were performed at 298.15 K. All the geometry optimization and energy calculations were performed considering the solvation effect for the sensitivity of solvation stability, which was applied using the polarizable continuum model (PCM). To be consistent with the universal solvation environment, two parameters were set for PCM, ε = 64.9 (which is the dielectric constant of PC) and epsinf = 2.5. B3LYP-D3 (BJ) was used to describe the van der Waals force, and the HOMO–LUMO calculations were performed at the B3LYP/6-311+g(d,p) level. NMR spectra were calculated under solvation environment and investigated using the module of NMR plotting in Multiwfn analysis [48]; the absorption peak of ^19^F is primarily considered. The RESP charge was also investigated by multiwfn. All calculations were performed on the Gaussian 16 package [49].

## 4. Conclusions

The fluorination of the cyclic ester molecule, EC, and the linear ester molecule, DME, were systematically investigated using the density functional theory calculation. For EC, we used -CF_1_ and -CF_2_ to completely replace the four H atoms of EC with F atoms step by step; for DME, we used the -CF_1_, -CF_2_, and -CF_3_ units. Varying their types, numbers, and positions, we investigated the regulating effect of fluorination on both solvent systems and studied the bond length/angle, VEA, RESP charge, and HOMO–LUMO. The NMR spectra revealed that the fully fluorinated cyclic molecule, tetra-FEC, as well as the more highly fluorinated linear molecules, CF_3_CF_1_CF_1_-, CF_3_CF_2_CF_2_-, CF_3_CF_1_CF_2_CF_3_, and CF_3_CF_2_CF_2_CF_3_, could dissolve Na-ion better and exhibit a larger ESW and higher reductive activity. That is, -CF_3_ is the most important group, and equally important are F atoms number and the way the groups are combined. If there is only one -CF_3_ group, fluorination with the same group in the adjacent positions is preferred; if there are two -CF_3_ groups, then any group in the adjacent positions has optimal performance for the solvent molecules have the largest number of F atoms at this time. The five fluorinated solvent molecules have a balanced and superior overall performance to other molecules of the same type. The design strategy of optimal SIB electrolyte fluorination represented in this study could be used to develop novel ideal electrolytes for energy store devices.

## Figures and Tables

**Table 1:** Bond lengths, bond angles of Na-ion, and fluorinated DME.

	Bond Length B_Na-O_(Å)	Bond Length B_Na-F_(Å)	Bond Length B_C-F_(Å)	Bond Angle α_O-Na-F_(deg)
-CF_1_	2.372	2.398	1.449	68.85
-CF_1_CF_1_	2.455	2.393	1.435	65.953
-CF_2_	2.38	2.536~3.593	1.380~1.403	66.086
-CF_1_CF_2_	2.421	2.654~3.650	1.376~1.398	63.719
-CF_2_CF_2_-	2.594	2.911~3.570	1.367~1.381	58.812
-CF_3_	2.984	3.465	1.340~1.356	38.426
CF_3_CF_1_-	3.133	3.497	1.334~1.349	37.692
CF_3_CH_2_CF_1_-	2.621	2.375~3.440	1.336~1.443	64.541
CF_3_CF_2_-	3.149	3.556	1.333~1.358	37.087
CF_3_CH_2_CF_2_-	2.742	2.518~3.469	1.334~1.400	60.688
CF_3_CF_1_CF_1_-	3.063	3.487~3.491	1.332~1.411	48.166
CF_3_CF_1_CF_2_-	2.690	2.480~3.713	1.328~1.394	61.661
CF_3_CF_2_CF_1_-	3.131	2.523~3.823	1.330~1.421	55.358
CF_3_CF_2_CF_2_-	3.230	3.432~3.512	1.329~1.371	47.691
CF_3_-CF_3_	3.082	3.501	1.355~1.337	37.789
CF_3_-CF_1_CF_3_	3.094	3.511~3.553	1.331~1.352	48.943
CF_3_-CF_2_CF_3_	3.187	3.425~3.524	1.330~1.356	48.926
CF_3_CF_1_CF_1_CF_3_	3.197	3.417~3.511	1.329~1.375	49.056
CF_3_CF_1_CF_2_CF_3_	3.225	3.428~3.524	1.327~1.351	48.489
CF_3_CF_2_CF_2_CF_3_	3.220	3.440~3.536	1.326~1.347	48.710

## Supplementary Materials

The following supporting information can be downloaded at: https://www.mdpi.com/article/10.3390/molecules27206949/s1, Figure S1: The electrostatic potential mapping of [Na^+^-solvent] complex of Tetra-FEC&Na (a), CF_3_CF_1_CF_1_&Na (b), CF_3_CF_2_CF_2_&Na (c), CF_3_CF_1_CF_2_CF_3_&Na (d), CF_3_CF_2_CF_2_CF_3_&Na; Table S1: Cartesian Coordinates of [Na^+^-Tetra-FEC]; Table S2: Cartesian Coordinates of [Na^+^-CF_3_CF_1_CF_1_-]; Table S3: Cartesian Coordinates of [Na^+^-CF_3_CF_2_CF_2_-]; Table S4: Cartesian Coordinates of [Na^+^-CF_3_CF_1_CF_2_CF_3_]; Table S5: Cartesian Coordinates of [Na^+^- CF_3_CF_2_CF_2_CF_3_].

## Abbreviations

SIB	Sodium-ion Battery
LIB	Lithium-ion Battery
SEI	Solid Electrolyte Interphase
EC	Ethylene Carbonate
FEC	Fluorinated Ethylene Carbonate
Trans-DFEC	Trans-Difluoroethylene Carbonates
Cis-DFEC	Cis-Difluoroethylene Carbonate
Tri-FEC	Trifluoroethylene Carbonate
Tetra-FEC	Tetrafluoroethylene Carbonate
FDMB	Fluorinated 1,4-Dimethoxybutane
DME	1,2-Dimethoxylethane
CE	Coulombic Efficiency
HOMO	Highest Occupied Molecular Orbital
LUMO	Lowest Unoccupied Molecular Orbital
RESP	Restrained Electrostatic Potential
VEA	Vertical Electron Affinity
ESW	Electrochemical Stability Window
NMR	Nuclear Magnetic Resonance
PC	Pearson Correlation
DFT	Density Function Theory
PCM	Polarizable Continuum Model

## References

[1] Pan H., Hu Y.-S., Chen L. (2013). Room-temperature stationary sodium-ion batteries for large-scale electric energy storage. Energy Environ. Sci..

[2] Sawicki M., Shaw L.L. (2015). Advances and challenges of sodium ion batteries as post lithium ion batteries. RSC Adv..

[3] Ponrouch A., Dedryvère R., Monti D., Demet A.E., Mba J.M.A., Croguennec L., Masquelier C., Johansson P., Palacín M.R. (2013). Towards high energy density sodium ion batteries through electrolyte optimization. Energy Environ. Sci..

[4] Goodenough J.B., Kim Y. (2010). Challenges for rechargeable Li batteries. Chem. Mater..

[5] Liu Y., Lin D., Li Y., Chen G., Pei A., Nix O., Li Y., Cui Y. (2018). Solubility-mediated sustained release enabling nitrate additive in carbonate electrolytes for stable lithium metal anode. Nat. Commun..

[6] Fan X., Chen L., Borodin O., Ji X., Chen J., Hou S., Deng T., Zheng J., Yang C., Liou S.-C. (2018). Non-flammable electrolyte enables Li-metal batteries with aggressive cathode chemistries. Nat. Nanotechnol..

[7] Fan X., Ji X., Chen L., Chen J., Deng T., Han F., Yue J., Piao N., Wang R., Zhou X. (2019). All-temperature batteries enabled by fluorinated electrolytes with non-polar solvents. Nat. Energy.

[8] Yamada Y., Wang J., Ko S., Watanabe E., Yamada A. (2019). Advances and issues in developing salt-concentrated battery electrolytes. Nat. Energy.

[9] Fan X., Chen L., Ji X., Deng T., Hou S., Chen J., Zheng J., Wang F., Jiang J., Xu K. (2018). Highly fluorinated interphases enable high-voltage Li-metal batteries. Chem.

[10] Qi S., Liu J., He J., Wang H., Wu M., Wu D., Huang J., Li F., Li X., Ren Y. (2021). Structurally tunable characteristics of ionic liquids for optimizing lithium plating/stripping via electrolyte engineering. J. Energy Chem..

[11] Shiraishi S., Kanamura K., Takehara Z. (1999). Surface Condition Changes in Lithium Metal Deposited in Nonaqueous Electrolyte Containing HF by Dissolution-Deposition Cycles. J. Electrochem. Soc..

[12] Zheng J., Engelhard M.H., Mei D., Jiao S., Polzin B.J., Zhang J.-G., Xu W. (2017). Electrolyte additive enabled fast charging and stable cycling lithium metal batteries. Nat. Energy.

[13] He J., Wang H., Zhou Q., Qi S., Wu M., Li F., Hu W., Ma J. (2021). Unveiling the role of Li+ solvation structures with commercial carbonates in the formation of solid electrolyte interphase for lithium metal batteries. Small Methods.

[14] Liang H.J., Gu Z.Y., Zhao X.X., Guo J.Z., Yang J.L., Li W.H., Li B., Liu Z.M., Li W.L., Wu X.L. (2021). Ether-Based Electrolyte Chemistry Towards High-Voltage and Long-Life Na-Ion Full Batteries. Angew. Chem. Int. Ed..

[15] Ren X., Chen S., Lee H., Mei D., Engelhard M.H., Burton S.D., Zhao W., Zheng J., Li Q., Ding M.S. (2018). Localized high-concentration sulfone electrolytes for high-efficiency lithium-metal batteries. Chem.

[16] Chen S., Zheng J., Mei D., Han K.S., Engelhard M.H., Zhao W., Xu W., Liu J., Zhang J.G. (2018). High-voltage lithium-metal batteries enabled by localized high-concentration electrolytes. Adv. Mater..

[17] Ding F., Xu W., Chen X., Zhang J., Engelhard M.H., Zhang Y., Johnson B.R., Crum J.V., Blake T.A., Liu X. (2013). Effects of carbonate solvents and lithium salts on morphology and coulombic efficiency of lithium electrode. J. Electrochem. Soc..

[18] Wang H., Yu Z., Kong X., Kim S.C., Boyle D.T., Qin J., Bao Z., Cui Y. (2022). Liquid electrolyte: The nexus of practical lithium metal batteries. Joule.

[19] Von Aspern N., Röschenthaler G.V., Winter M., Cekic-Laskovic I. (2019). Fluorine and lithium: Ideal partners for high-performance rechargeable battery electrolytes. Angew. Chem. Int. Ed..

[20] Yu Z., Rudnicki P.E., Zhang Z., Huang Z., Celik H., Oyakhire S.T., Chen Y., Kong X., Kim S.C., Xiao X. (2022). Rational solvent molecule tuning for high-performance lithium metal battery electrolytes. Nat. Energy.

[21] Zhang Y., Viswanathan V. (2020). Not All fluorination is the same: Unique effects of fluorine functionalization of ethylene carbonate for tuning solid-electrolyte interphase in Li metal Batteries. Langmuir.

[22] Vogt L.O., El Kazzi M., Jämstorp Berg E., Pérez Villar S.A., Novak P., Villevieille C. (2015). Understanding the interaction of the carbonates and binder in Na-ion batteries: A combined bulk and surface study. Chem. Mater..

[23] Yu Z., Yu W., Chen Y., Mondonico L., Xiao X., Zheng Y., Liu F., Hung S.T., Cui Y., Bao Z. (2022). Tuning Fluorination of Linear Carbonate for Lithium-Ion Batteries. J. Electrochem. Soc..

[24] Han J.-G., Lee J.B., Cha A., Lee T.K., Cho W., Chae S., Kang S.J., Kwak S.K., Cho J., Hong S.Y. (2018). Unsymmetrical fluorinated malonatoborate as an amphoteric additive for high-energy-density lithium-ion batteries. Energy Environ. Sci..

[25] Liang H.-J., Gu Z.-Y., Zhao X.-X., Guo J.-Z., Yang J.-L., Li W.-H., Li B., Liu Z.-M., Sun Z.-H., Zhang J.-P. (2022). Advanced flame-retardant electrolyte for highly stabilized K-ion storage in graphite anode. Sci. Bull..

[26] Bolloli M., Alloin F., Kalhoff J., Bresser D., Passerini S., Judeinstein P., Leprêtre J.-C., Sanchez J.-Y. (2015). Effect of carbonates fluorination on the properties of LiTFSI-based electrolytes for Li-ion batteries. Electrochim. Acta.

[27] He M., Su C.-C., Peebles C., Zhang Z. (2021). The impact of different substituents in fluorinated cyclic carbonates in the performance of high voltage lithium-ion battery electrolyte. J. Electrochem. Soc..

[28] Yu Z., Wang H., Kong X., Huang W., Tsao Y., Mackanic D.G., Wang K., Wang X., Huang W., Choudhury S. (2020). Molecular design for electrolyte solvents enabling energy-dense and long-cycling lithium metal batteries. Nat. Energy.

[29] Zheng X., Gu Z., Liu X., Wang Z., Wen J., Wu X., Luo W., Huang Y. (2020). Bridging the immiscibility of an all-fluoride fire extinguishant with highly-fluorinated electrolytes toward safe sodium metal batteries. Energy Environ. Sci..

[30] Wu S., Su B., Ni K., Pan F., Wang C., Zhang K., Yu D.Y., Zhu Y., Zhang W. (2021). Fluorinated Carbonate Electrolyte with Superior Oxidative Stability Enables Long-Term Cycle Stability of Na2/3Ni1/3Mn2/3O2 Cathodes in Sodium-Ion Batteries. Adv. Energy Mater..

[31] Cresce A.V., Russell S.M., Borodin O., Allen J.A., Schroeder M.A., Dai M., Peng J., Gobet M.P., Greenbaum S.G., Rogers R.E. (2017). Solvation behavior of carbonate-based electrolytes in sodium ion batteries. Phys. Chem. Chem. Phys..

[32] Liu X., Zheng X., Dai Y., Wu W., Huang Y., Fu H., Huang Y., Luo W. (2021). Fluoride-Rich Solid-Electrolyte-Interface Enabling Stable Sodium Metal Batteries in High-Safe Electrolytes. Adv. Funct. Mater..

[33] Li Q., Cao Z., Wahyudi W., Liu G., Park G.-T., Cavallo L., Anthopoulos T.D., Wang L., Sun Y.-K., Alshareef H.N. (2020). Unraveling the new role of an ethylene carbonate solvation shell in rechargeable metal ion batteries. ACS Energy Lett..

[34] Chen X., Bai Y.K., Zhao C.Z., Shen X., Zhang Q. (2020). Lithium bonds in lithium batteries. Angew. Chem..

[35] Wang C., Wu J., Zhao X., Wang L., Yin Z. (2021). Lithium Bond-Enhanced Capacity of Dipyridyl Polysulfides for LSBs. ACS Appl. Energy Mater..

[36] Shakourian-Fard M., Kamath G., Smith K., Xiong H., Sankaranarayanan S.K. (2015). Trends in Na-ion solvation with alkyl-carbonate electrolytes for sodium-ion batteries: Insights from first-principles calculations. J. Phys. Chem. C.

[37] Zhu S., Chen J. (2022). Dual strategy with Li-ion solvation and solid electrolyte interphase for high Coulombic efficiency of lithium metal anode. Energy Storage Mater..

[38] Ren X., Gao P., Zou L., Jiao S., Cao X., Zhang X., Jia H., Engelhard M.H., Matthews B.E., Wu H. (2020). Role of inner solvation sheath within salt–solvent complexes in tailoring electrode/electrolyte interphases for lithium metal batteries. Proc. Natl. Acad. Sci. USA.

[39] Chen X., Shen X., Li B., Peng H.J., Cheng X.B., Li B.Q., Zhang X.Q., Huang J.Q., Zhang Q. (2018). Ion–solvent complexes promote gas evolution from electrolytes on a sodium metal anode. Angew. Chem. Int. Ed..

[40] Wang E., Niu Y., Yin Y.-X., Guo Y.-G. (2020). Manipulating electrode/electrolyte interphases of sodium-ion batteries: Strategies and perspectives. ACS Mater. Lett..

[41] Li Y., Wu F., Li Y., Liu M., Feng X., Bai Y., Wu C. (2022). Ether-based electrolytes for sodium ion batteries. Chem. Soc. Rev..

[42] Xing L., Zheng X., Schroeder M., Alvarado J., von Wald Cresce A., Xu K., Li Q., Li W. (2018). Deciphering the ethylene carbonate–propylene carbonate mystery in Li-ion batteries. Acc. Chem. Res..

[43] Komaba S., Ishikawa T., Yabuuchi N., Murata W., Ito A., Ohsawa Y. (2011). Fluorinated ethylene carbonate as electrolyte additive for rechargeable Na batteries. ACS Appl. Mater. Interfaces.

[44] Pham T.A., Kweon K.E., Samanta A., Lordi V., Pask J.E. (2017). Solvation and dynamics of sodium and potassium in ethylene carbonate from ab initio molecular dynamics simulations. J. Phys. Chem. C.

[45] Xu M., Li Y., Ihsan-Ul-Haq M., Mubarak N., Liu Z., Wu J., Luo Z., Kim J.K. (2022). NaF-rich solid electrolyte interphase for dendrite-free sodium metal batteries. Energy Storage Mater..

[46] Liao Q., Mu M., Zhao S., Zhang L., Jiang T., Ye J., Shen X., Zhou G. (2017). Performance assessment and classification of retired lithium ion battery from electric vehicles for energy storage. Int. J. Hydrogen Energy.

[47] Zhao Y., Zhou T., Ashirov T., Kazzi M.E., Cancellieri C., Jeurgens L.P., Choi J.W., Coskun A. (2022). Fluorinated ether electrolyte with controlled solvation structure for high voltage lithium metal batteries. Nat. Commun..

[48] Lu T., Chen F. (2012). Multiwfn: A multifunctional wavefunction analyzer. J. Comput. Chem..

[49] Frisch M.J., Trucks G.W., Schlegel H.B., Scuseria G.E., Robb M.A., Cheeseman J.R., Scalmani G., Barone V., Petersson G.A., Nakatsuji H. (2016). Gaussian 16 Rev. C.01.

