# Supplementary material for: First-Principles-Based Optimized Design of Fluoride Electrolytes for Sodium-Ion Batteries
Source: Molecules. 2022 Oct 17;27(20):6949. doi: 10.3390/molecules27206949 (PMC9611088; doi:10.3390/molecules27206949)
Supplement: Supplementary file 1 [file molecules-27-06949-s001.zip › molecules-1948231-supplementary.pdf]

Supplementary Materials

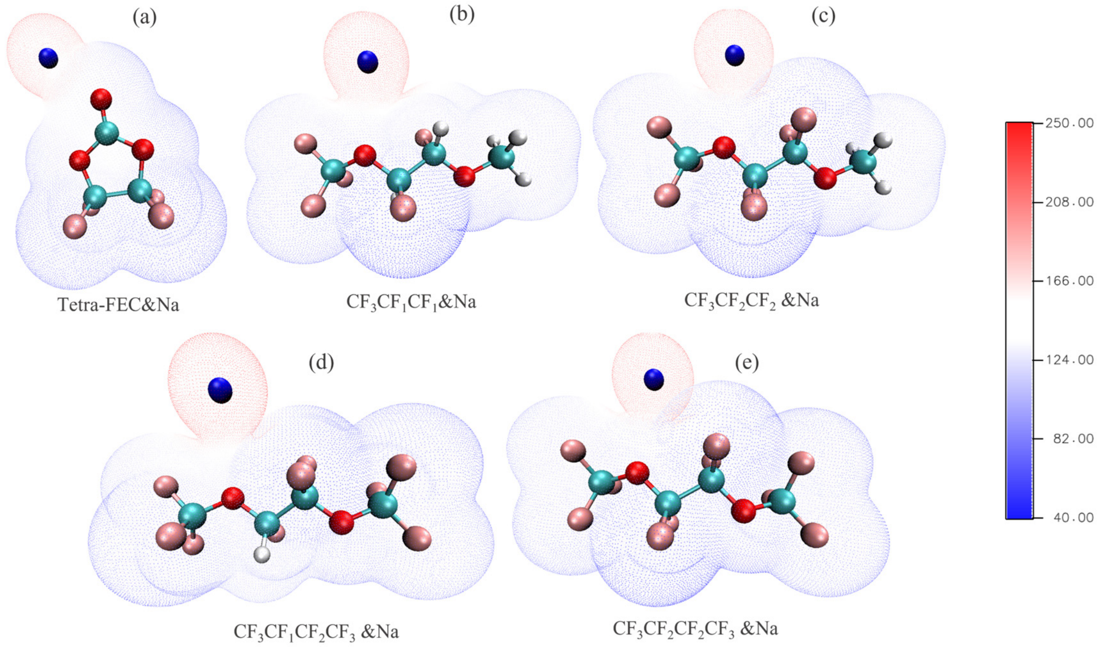

FigureS1. The electrostatic potential mapping of [Na^+-solvent] complex of Tetra-FEC&Na(**a**), $CF_3CF_1CF_1$&Na(**b**), $CF_3CF_2CF_2$&Na(**c**), $CF_3CF_1CF_2CF_3$&Na(**d**), $CF_3CF_2CF_2CF_3$&Na(**e**)

Table S1. Cartesian Coordinates of [Na^+-Tetra-FEC]

Center Number	Atomic Name	Coordinates (Angstroms)		
		x	y	z
1	O	-0.514	0.809	0
2	O	0.105	-1.343	0
3	O	-2.083	-0.812	0
4	C	0.878	0.862	0
5	C	1.312	-0.649	0
6	C	-0.946	-0.481	0
7	F	1.306	1.511	1.083
8	F	1.306	1.511	-1.083
9	F	2.019	-0.971	-1.083
10	F	2.019	-0.971	1.083
11	Na	-4.307	0.242	0

Table S2. Cartesian Coordinates of [Na^{+}-$CF_3CF_1CF_1$-]

Center Number	Atomic Name	Coordinates (Angstroms)		
		x	y	z
1	C	1.964	-0.39	0.178
2	O	0.758	0.077	-0.232
3	C	-0.349	-0.794	-0.15
4	C	-1.599	0.068	0.017
5	O	-2.66	-0.766	0.157
6	C	-3.95	-0.137	0.024
7	F	2.855	0.583	-0.001
8	F	1.959	-0.734	1.479
9	F	2.363	-1.471	-0.516
10	H	-0.242	-1.512	0.662
11	F	-0.451	-1.484	-1.338
12	H	-1.708	0.781	-0.807
13	F	-1.411	0.84	1.183
14	H	-4.681	-0.94	0.045
15	H	-4.126	0.549	0.854
16	H	-4.011	0.398	-0.927
17	Na	0.524	3.105	-0.628

Table S3. Cartesian Coordinates of [Na^{+}-$CF_3CF_2CF_2$-]

Center Number	Atomic Name	Coordinates (Angstroms)		
		x	y	z
1	C	2.14	-0.304	0.046
2	O	0.849	0.104	-0.199
3	C	-0.21	-0.784	-0.131
4	C	-1.516	0.054	-0.005
5	O	-2.551	-0.777	0.041
6	C	-3.871	-0.176	0.178
7	F	2.933	0.662	-0.408
8	F	2.366	-0.463	1.354
9	F	2.442	-1.45	-0.573
10	F	-0.097	-1.615	0.924
11	F	-0.264	-1.545	-1.243
12	F	-1.574	0.925	-1.062
13	F	-1.412	0.844	1.11
14	H	-4.56	-1.015	0.173
15	H	-3.934	0.364	1.121
16	H	-4.067	0.484	-0.666
17	Na	0.67	3.326	-0.073

Table S4. Cartesian Coordinates of [Na^+- $CF_3CF_1CF_2CF_3$]

Center Number	Atomic Name	Coordinates (Angstroms)		
		x	y	z
1	C	3.075	-0.166	0.119
2	O	1.81	-0.674	0.31
3	C	0.687	0.095	0.07
4	C	-0.504	-0.878	-0.032
5	O	-1.629	-0.098	-0.318
6	C	-2.833	-0.526	0.162
7	F	3.911	-0.976	0.757
8	F	3.403	-0.146	-1.177
9	F	3.207	1.074	0.602
10	F	0.811	0.828	-1.056
11	F	0.472	0.957	1.088
12	H	-0.606	-1.448	0.891
13	F	-0.273	-1.739	-1.069
14	F	-3.761	0.307	-0.293
15	F	-3.125	-1.771	-0.242
16	F	-2.863	-0.521	1.505
17	Na	-1.766	3.124	-0.343

Table S5. Cartesian Coordinates of [Na^+- $CF_3CF_2CF_2CF_3$]

Center Number	Atomic Name	Coordinates (Angstroms)		
		x	y	z
1	C	2.843	-0.409	0.108
2	O	1.595	0.066	-0.247
3	C	0.485	-0.749	-0.209
4	C	-0.76	0.193	-0.195
5	O	-1.868	-0.62	-0.259
6	C	-3.115	-0.147	0.103
7	F	3.724	0.464	-0.361
8	F	2.973	-0.483	1.435
9	F	3.091	-1.615	-0.409
10	F	0.464	-1.531	0.884
11	F	0.432	-1.548	-1.29
12	F	-0.704	1.028	-1.251
13	F	-0.739	0.942	0.922
14	F	-4	-1.006	-0.382
15	F	-3.247	-0.092	1.431
16	F	-3.358	1.07	-0.393
17	Na	1.612	3.277	-0.006
